# Hypothyroidism in hibernating brown bears

**DOI:** 10.1186/s13044-022-00144-2

**Published:** 2023-02-01

**Authors:** Anne Mette Frøbert, Claus G. Nielsen, Malene Brohus, Jonas Kindberg, Ole Fröbert, Michael T. Overgaard

**Affiliations:** 1 Department of Chemistry and Bioscience, Faculty of Engineering and Science, Aalborg University, Fredrik Bajers Vej 7H, 9220 Aalborg East, Denmark; 2 Department of Clinical Biochemistry, Aalborg University Hospital, Aalborg, Denmark; 3 Department of Wildlife, Fish and Environmental Studies, Swedish University of Agricultural Sciences, Umeå, Sweden; 4 Norwegian Institute for Nature Research, Trondheim, Norway; 5 Steno Diabetes Center Aarhus, Aarhus University Hospital, Aarhus, Denmark; 6 Department of Cardiology, Faculty of Health, Örebro University, Örebro, Sweden; 7 Department of Clinical Medicine, Faculty of Health, Aarhus University, Aarhus, Denmark; 8 Department of Clinical Pharmacology, Aarhus University Hospital, Aarhus, Denmark

**Keywords:** Thyroid hormone, Thyroxine, Triiodothyronine, Thyroxine-binding globulin (TBG), *Ursus arctos*, Hibernation, Hypothyroidism, Metabolism

## Abstract

Brown bears hibernate throughout half of the year as a survival strategy to reduce energy consumption during prolonged periods with scarcity of food and water. Thyroid hormones are the major endocrine regulators of basal metabolic rate in humans. Therefore, we aimed to determine regulations in serum thyroid hormone levels in hibernation compared to the active state to investigate if these are involved in the adaptions for hibernation.

We used electrochemiluminescence immunoassay to quantify total triiodothyronine (T_3_) and thyroxine (T_4_) levels in hibernation and active state in paired serum samples from six subadult Scandinavian brown bears. Additionally, we determined regulations in the liver mRNA levels of three major thyroid hormone-binding proteins; thyroxine-binding globulin (TBG), transthyretin (TTR), and albumin, by analysis of previously published grizzly bear RNA sequencing data*.*

We found that bears were hypothyroid when hibernating with T_4_ levels reduced to less than 44% (*P* = 0.008) and T_3_ levels reduced to less than 36% (*P* = 0.016) of those measured in the active state. In hibernation, mRNA levels of TBG and albumin increased to 449% (*P* = 0.031) and 121% (*P* = 0.031), respectively, of those measured in the active state. TTR mRNA levels did not change.

Hibernating bears are hypothyroid and share physiologic features with hypothyroid humans, including decreased basal metabolic rate, bradycardia, hypothermia, and fatigue. We speculate that decreased thyroid hormone signaling is a key mediator of hibernation physiology in bears. Our findings shed light on the translational potential of bear hibernation physiology to humans for whom a similar hypometabolic state could be of interest in specific conditions.

## Introduction

The Scandinavian brown bear is physically inactive throughout half of the year while hibernating as a survival strategy to reduce energy consumption during prolonged periods with scarcity of food and water. In this period, bears do not eat, drink, urinate, or defecate [1]. In the hibernation period, bears exclusively survive from consumption of fat stored in adipose tissue [1] and they lower metabolic rate to 25% of the active state [2]. In late summer, the bears become hyperphagic to accumulate a considerable fat reserve in preparation for the hibernation period. As fat serves as the primary metabolic fuel, lean body mass is conserved during hibernation [3–5].

Thyroid hormones are the major endocrine regulators of metabolic rate in humans [6]. In this study we therefore aimed to map the regulations of thyroid hormone levels in bear serum in hibernation and active state to elucidate their potential involvement in the adaptions for hibernation.

Thyroxine (T_4_) is the major circulating thyroid hormone in humans. However, triiodothyronine (T_3_) is considered the active form due to its 15-fold higher affinity for the thyroid receptors compared to T_4_. The conversion of inactive T_4_ to active T_3_ is reversible and mediated by deiodinases in various tissues [7, 8]. The thyroid hormones are lipophilic and the majority (> 99%) are therefore bound to different transport proteins in the circulation, including thyroxine-binding globulin (TBG, alternative name SerpinA7), transthyretin (TTR), and albumin, with TBG being the major thyroid hormone carrier [9]. Like most plasma proteins, these transport proteins are all expressed in the liver. Although it was originally believed that only unbound free thyroid hormones are available for cellular uptake through passive diffusion, tissue-specific transporter proteins promoting hormonal cellular uptake of thyroid hormones have been reported [6, 10]. Additionally, proteolytic cleavage of the binding proteins locally in the tissues has been suggested to reduce hormone affinity and thereby facilitate thyroid hormone delivery [11].

## Methods

### Serum samples

Arterial blood was collected in serum collection tubes (BD Vacutainer®) from free-ranging (wild) Scandinavian Brown Bears (*Ursus arctos arctos*) in Dalarna, Central Sweden in hibernation (February) and active state (June). The hibernation period generally lasted from October to April. Samples from 6 subadult 2- to 3-year-old (y/o) Scandinavian brown bears (3 males and 3 females) were analyzed in this study (Table 1). The females were sampled for two consecutive years at both 2 and 3 y. The male bears were too heavy to handle at age 3. The blood samples were kept at 5 °C until centrifugation at 200 g for 15 min, immediately upon returning from the field after 1–2 h. Thereafter, serum was stored at -80 °C. Details on the methods of bear capture, anesthesia, and blood sample collection have been published previously [12]. In brief, bears were marked with GPS collars and VHF transmitters to enable localization of the bears. When hibernating, the bears were located in their dens and anaesthetized with a mixture of medetomidine, zolazepam, tiletamine, and ketamine. In the active state, the same bears were located in their habitat and darted from a helicopter with a mixture of medetomidine, zolazepam, and tiletamine. Anesthesia was antagonized by atipamezole. In winter, the bears were placed back into their dens. Captures were performed by The Scandinavian Brown Bear Research Project (http://bearproject.info/).

**Table 1:** Serum samples analyzed in this study

Bear ID	Gender	2 y/o		3 y/o	
		Hibernation	Active state	Hibernation	Active state
W1802	Male	W19	S19		
W1814	Male	W19	S19		
W1910	Male	W20	S20		
W1509	Female	W16	S16	W17	S17
W1806	Female	W19	S19	W20	S20
W1813	Female		S19	W20	S20

The internal bear IDs applied in the Scandinavian Brown Bear Research Project are stated in the first column (Wxxxx). The numbers in columns 3 to 6 indicate the sampling year, e.g. W17 is a hibernation (winter) sample from 2017 and S17 is an active state (summer) sample from 2017

### Thyroid hormone measurements

Total T_4_ and T_3_ levels in the serum samples were measured by electrochemiluminescence immunoassay (ECLIA) using the Elecsys® T_3_ and Elecsys® T_4_ assays in combination with the Roche cobas 8000 e602 Immunoassay Analyzer according to the manufacturer’s instructions (Roche Diagnostics). Technical replicates were not performed, as the intra-assay coefficient of variance is below 5%.

### RNA-seq analysis of RNA sequencing data

The RNA sequencing data generated by Jansen et al. (Genbank BioProject PRJNA413091) from liver tissue of six adult captive grizzly bears (2 females, 4 males, ranging from 5 to 13 years old) in hibernation (January) and the active state (May) were analyzed [13]. The hibernation period for the captive bears lasted from early November to mid-March [13].

The sequencing data were processed using CLC Genomics Workbench 20.0: The data were trimmed using Trim Reads 2.4 and applying automatic read-through adapter trimming, a quality score limit of 0.05, and an ambiguous limit of two. RNA-Seq Analysis 2.2 was performed on the trimmed reads with mapping to the *Ursus arctos horribilis* reference genome assembly (GCF_003584765.1) with annotated transcripts (mismatch cost = 2, insertion cost = 3, deletion cost = 3, and length fraction = 0.8).

### Data analysis

Fold-changes (FCs) were calculated from the average of the log_2_ transformed within-animal comparisons. For datasets with values below the detection limit, medians and FCs were calculated by substituting the measured value (if below the detection limit) with the detection limit of the assay. The detection limits of the applied analysis methods were 0.30 nM for T_3_ and 5.40 nM for T_4_. Data are presented as median (range).

Statistical analysis was performed using a Wilcoxon matched-pairs signed rank test, since most thyroid hormone measurements were below the detection limit in the winter samples. Measurements below the detection limit were set to the lowest rank in the statistical analysis. *P* < 0.05 was regarded as significant.

## Results

The findings in this study are summarized in Table 2. Total thyroid hormone concentrations, representing the sum of free and protein-bound hormone, were measured in serum from 6 bears (3 males and 3 females) in hibernation and active state, and for some bears during two seasons.

**Table 2:** Regulations in thyroid hormone levels and in mRNA levels of the major thyroid hormone-binding proteins

			Winter/summer ratio Median (range) of paired samples			
	Active state (nM) Median (range)	Hibernation (nM) Median (range)	Log_2_(FC)	FC (%)	*P*	Method
T_4_	13.6 (7.39 to 19.76)	< 5.4 (< 5.4 to 9.68)	-1.20 (-1.87 to -0.15)	44* (27 to 90)	0.008	Immuno-assay
T_3_	0.82 (0.68 to 1.06)	< 0.3 (< 0.3 to < 0.3)	-1.46 (-1.18 to -1.82)	36* (28 to 44)	0.016	Immuno-assay
TBG	−	−	2.17 (1.24 to 6.37)	449* (237 to 8243)	0.031	RNA-seq
TTR	−	−	0.49 (-0.26 to 0.56)	140 (84 to 147)	0.156	RNA-seq
Albumin	−	−	0.27 (0.17 to 0.53)	121* (113 to 144)	0.031	RNA-seq

Summary of regulations in the thyroid hormone levels in brown bear serum samples and in the mRNA levels of the major thyroid hormone-binding proteins in grizzly bear liver samples from active state and hibernation. *Significant difference (*P* < 0.05)

**Fig. 1:**
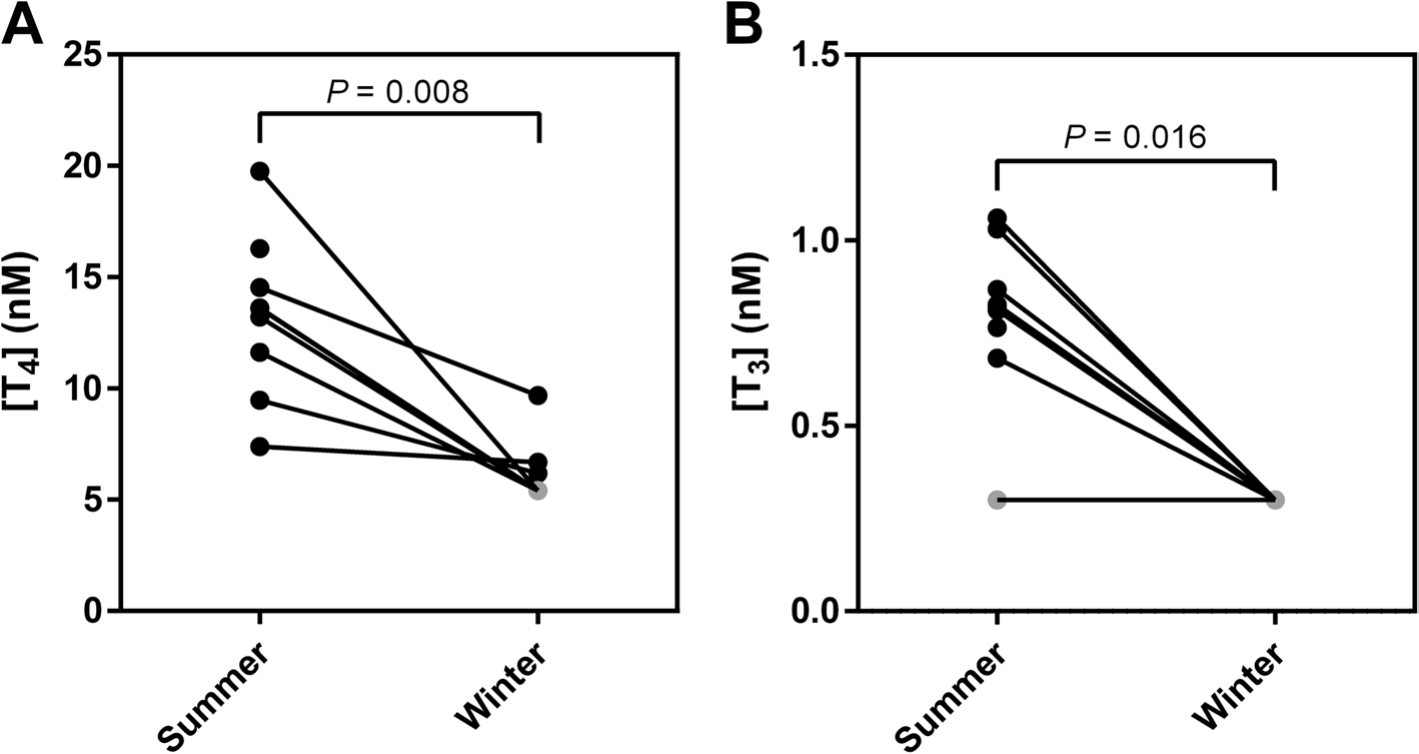
Thyroid hormone levels. Concentrations of **A** thyroxine (T_4_) and **B** triiodothyronine (T_3_) in paired serum samples from active (summer) and hibernating (winter) bears quantified by electrochemiluminescence immunoassay. For measurements below the detection limit, the detection limit is plotted in grey

In hibernation, T_4_ was detected in three of eight samples, while it was below the detection limit of 5.4 nM in the remaining bears – thus the median T_4_ level in hibernation was below 5.4 nM (< 5.4 nM to 9.68 nM). This was significantly lower than the median serum T_4_ level in the active state of 13.6 nM (7.39 nM to 19.76 nM) (*P* = 0.008) (Fig. 1A).

Serum T_3_ levels were below the detection limit of 0.3 nM in all samples in hibernation, and significantly lower than the median active state level of 0.82 nM (0.68 nM to 1.06 nM) (excluding one sample with no detected T_3_) (*P* = 0.016) (Fig. 1B). Thus, thyroid hormone levels in hibernation were reduced to less than 44% (T_4_) and 36% (T_3_) of that measured in the active state. T_4_ and T_3_ levels did not differ between male and female bears.

**Fig. 2:**
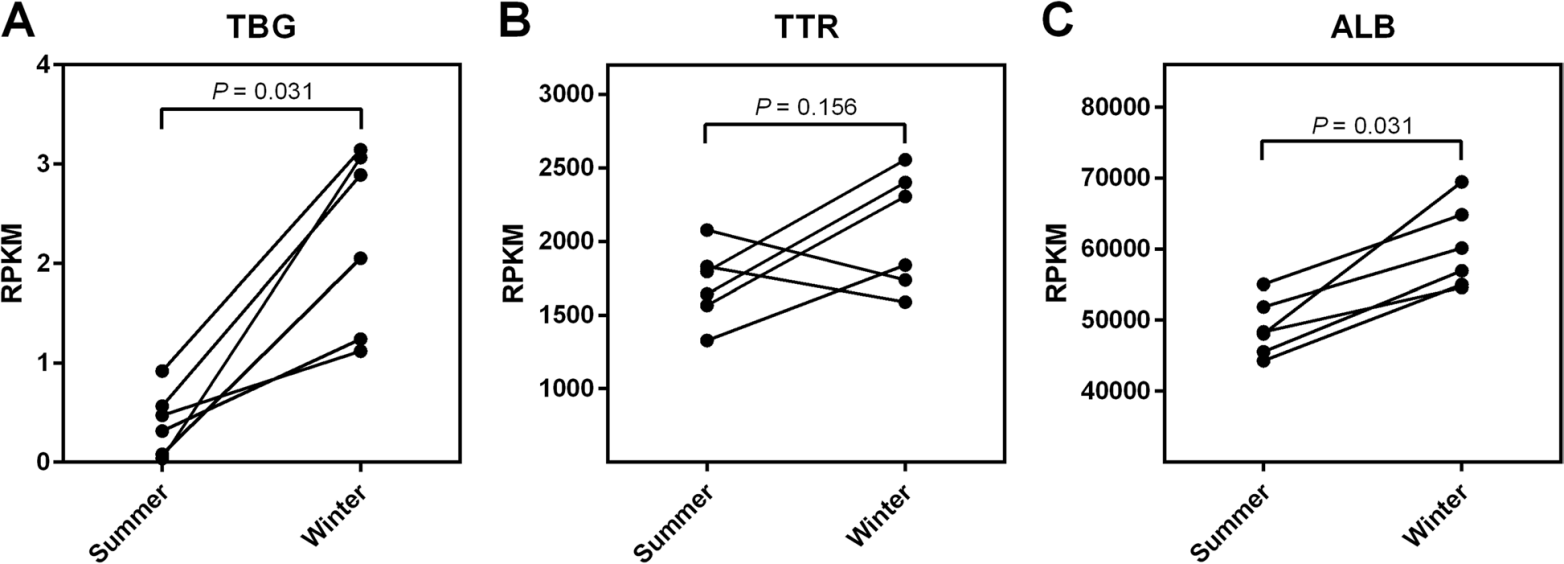
Liver mRNA levels of the major thyroid hormone-binding proteins. Normalized mRNA levels in Reads Per Kilobase of transcript per Million mapped reads (RPKM) of **A** thyroxine-binding globulin (TBG), **B** transthyretin (TTR), and **C** albumin (ALB) expressed in grizzly bear liver in active state (summer) and hibernation (winter), based on analysis of RNA-sequencing data from Jansen et al. [13]

In a reanalysis of liver transcript data from grizzly bears (n = 6) [13], a brown bear subspecies, we found that the median mRNA level of the major thyroid hormone carrier TBG increased to 449% in hibernation compared to the active state (*P* = 0.031) (Fig. 2A). No difference in the mRNA level of TTR was observed between hibernation and active state (FC = 140%, *P* = 0.156) (Fig. 2B), while the mRNA level of albumin increased to 121% in hibernation (*P* = 0.031) (Fig. 2C).

## Discussion

Regulations of the thyroid hormone levels in hibernating bears have previously been described [14–18], but to our awareness, this is the first study using paired free-ranging (wild) bears. Additionally, the knowledge of thyroid hormone function in humans has greatly expanded since the previous studies on bears were published, enabling us to discuss the biological effects of the thyroid hormone differences observed in bears in an updated context. Thyroid hormones are present in all vertebrates, where they affect metabolic activity, growth, and development, thereby supporting preservation of their functions across species [19].

Moreover, liver RNA sequencing data from grizzly bears have been published and the primary data are available [13]. These data enabled us to determine regulations in the transcript levels of thyroid hormone-carrier proteins. Liver samples were not collected from the wild Scandinavian brown bears due to concerns for internal bleeding after liver biopsy. Therefore, the analyses of thyroid hormone carrier protein mRNA levels are based on another brown bear subspecies, namely captive adult grizzly bears. Of note, the grizzly bears are not age matched to the sub-adult brown bears sampled in this study. Yet, we do not expect the hibernation mechanisms to differ between the two bear subspecies, and hibernation occurs in bears of all ages.

We found that the thyroid hormones, T_4_ (the most abundant) and T_3_ (the most potent), decreased during hibernation in Scandinavian brown bears in agreement with the results from most previous publications, which generally report decreases in both total and free thyroid hormone levels [14–18]. One study, however, found increased thyroid hormone levels in wild hibernating bears [20]. Paired samples were not examined in this study, which limits the statistical power of the analyses, especially due to the small sample size of 4 to 5 bears, and in light of the large biological inter-individual variation observed in this study.

Thyroid hormone levels have been reported to drop by the onset of hibernation and to remain low throughout the hibernation period supporting that their regulation is related to hibernation [15]. Both the wild and the captive bears included in this study were sampled approximately in the middle of the hibernation period and approximately two months after terminating hibernation.

The bioavailability of the thyroid hormones is regulated by the three carrier proteins TBG, TTR, and albumin. In the grizzly bear liver transcript data by Jansen et al. [13], we observed that the mRNA level of TBG, the major thyroid hormone carrier, increased to ~ 450% in hibernation compared to the active state. TBG was not identified in two mass spectrometry-based proteomics studies from our group (ProteomeXchange datasets PXD003946 and PXD030482) despite being included in the database used for protein identification [21, 22]. Whether this is due to low plasma concentrations, post-translational modifications, or due to poor peptide ionization efficiency is not known. No difference in the TTR mRNA level was observed in agreement with the two proteomics studies [21, 22]. The albumin mRNA level increased to ~ 120% during hibernation in agreement with previously measured protein levels [13, 21, 23].

The transcript data by Jansen et al. [13] is based on captive bears. Many captive bears do not experience a long and continuous hibernation period, which may be due to disturbances or food availability not reflecting that of nature. Thus, the hibernation physiology of captive bears may differ from that of wild bears. Jansen et al., however, reports that food availability was increased during the hyperphagic period and discontinued during hibernation. Temperature and light conditions were also as in nature. The captive bears hibernated a little more than four months [13], which is within the normal range for wild bears, despite being shorter than the hibernation period of approximately five months for the wild bears in this study. Thus, although the study by Jansen et al. is based on bears in captivity, we believe that the data is translatable to wild bears as the surrounding conditions mimic those in nature.

In humans, the main function of TBG is to maintain stable thyroid hormone levels in the circulation. Free thyroid hormone levels are not affected by an increase in the TBG concentration as the higher binding capacity of TBG is offset by a prolonged half-life of the bound thyroid hormones, increasing the total thyroid hormone concentration. Vice versa, decreased TBG levels result in decreased total thyroid hormone levels due to a shorter half-life, again with the free thyroid hormone levels remaining constant as the binding capacity of TBG is also decreased [24]. In brown bears, however, the large increase in the TBG transcript level in combination with the large decrease in the total thyroid hormone levels likely results in a decrease in the free, bioactive thyroid hormone levels. This strongly indicates that bears are hypothyroid in hibernation. Since the decreased thyroid hormone levels in hibernation do not seem to be due to a decreased half-life, as the binding capacity of TBG appears to be increased, we suggest that the enzymatic synthesis in the thyroid gland is reduced [25]. To our awareness, no other functions of TBG than being a thyroid hormone regulator is known. Thus, the fact that a ~ 450% increase in the TBG mRNA level is necessary even though the thyroid hormone levels is reduced to less than 50% of active state supports that hypothyroidism is essential to bear hibernation.

**Fig. 3:**
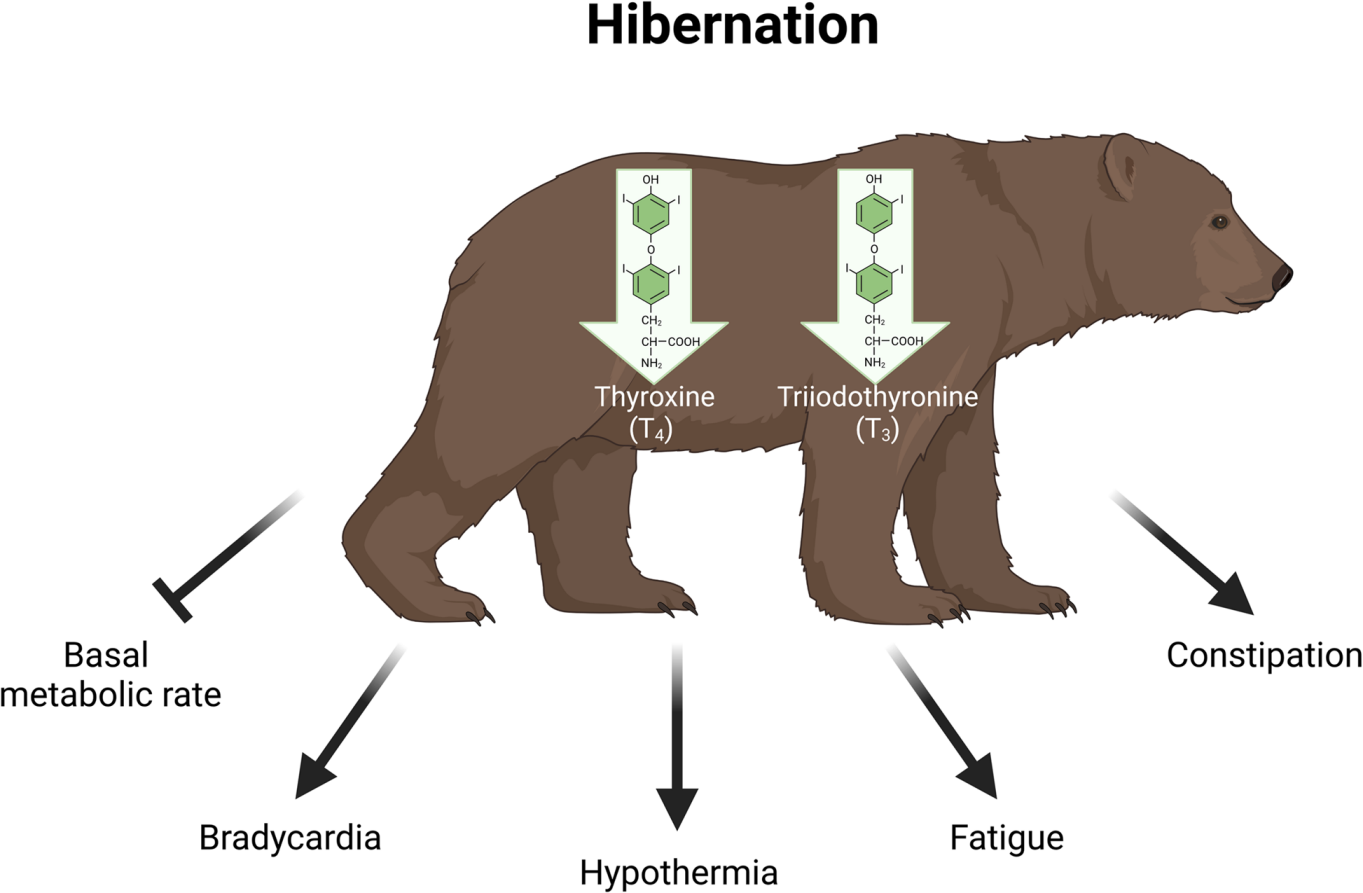
Shared physiology between hypothyroid humans and hibernating bears. Bears are hypothyroid during hibernation. Hibernating bears share physiology with hypothyroid humans, including decreased basal metabolic rate, bradycardia, hypothermia, fatigue, and constipation, and we therefore speculate that hypothyroidism is a key regulator of hibernation in bears. Created with BioRender.com.

Hypothyroidism in humans is typically associated with decreased basal metabolic rate, bradycardia (slower heart rate), hypothermia, constipation, and fatigue [7], and severe hypothyroidism in humans has been reported to lower the total body energy expenditure by as much as 50% [6]. A similar physiology is seen in hibernating bears with a metabolic rate reduced to 25% of that in the active state [1], slower heart rate [26, 27], decreased body temperature [4, 16], and no defecation [1]. Based on this similarity, we suggest that hypothyroidism is a key regulator of hibernation physiology in bears (Fig. 3).

In a human bed rest study, supplementation of low-dose T_3_ impaired nitrogen balance, lean body mass, protein breakdown, and bone resorption, supporting that a low T_3_ plasma level is preferable during bed rest to reduce muscle and bone catabolism [28, 29].

Thyroid hormones are key regulators of basal metabolic rate [6]. Caloric restriction or anorexia in humans lead to reduced thyroid hormone levels, which has been proposed to protect energy stores [8]. Bed rest, on the other hand, has not been found to alter the metabolic clearance of T_4_ and T_3_ in humans [30]. The decreased thyroid hormone levels in hibernating bears are therefore likely a consequence of the caloric restriction, contributing to the protective hibernation physiology.

Bear hibernation physiology has translational potential to humans in many different aspects. Despite severe obesity in the fall and inactivity for up to six months during hibernation, bears show no signs of harmful effects. Bears preserve insulin sensitivity during hyperphagia although gaining up to 30% body mass [31, 32]. During hibernation, bears become insulin resistant but remain euglycemic [32]. Despite a decreased heart rate, resulting in a low blood flow, hibernating bears show no signs of thrombosis [33]. Moreover, circulating cholesterol levels increase during hibernation and are much higher than normal physiological levels in humans for whom high cholesterol is associated with oxidative stress and inflammation, leading to necrosis, fibrosis, and calcification. Yet, hibernating bears do not develop atherosclerosis, and inflammatory markers decrease during hibernation [34, 35]. Bears lose less than 23% of their muscle strength over a hibernation period of 130 days, while humans at complete rest are predicted to lose 90% over the same period [36]. Moreover, bone density is preserved in hibernating bears, although mechanical unloading of bone is associated with osteoporosis in many other mammalian species including humans [37].

The mechanisms that drive hibernation physiology in bears may be used to address the detrimental consequences of obesity and inactivity in humans [38]. Introduction of a hypometabolic state may have relevance for treatment of disease-related weight loss (cachexia), such as that observed in cancer patients [39], or during space missions, where food and oxygen resources are limited [40].

### Strengths and limitations

In this study, we analyzed serum from subadult bears. The use of sexually immature animals reduced the risk of interference from pregnancy, sexual activity, and past diseases in the analyses. Thyroid hormone levels decline during childhood in humans [41], however, the observed regulations in thyroid hormone levels in this study are not expected to be age related, as similar regulations were observed in samples from two female bears collected for two consecutive years.

The dataset analyzed in this study is relatively small (*n* = 6) due to the difficulties obtaining samples from large wild animals. Paired winter and summer samples were collected to increase the statistical power of the analyses.

We measured thyroid hormone levels in Scandinavian brown bears and analyzed RNA sequencing data from another brown bear subspecies, American brown bears (grizzly bears). We do not expect this to affect the outcome of this study as the species are closely related.

Acute stress is a known cause of decreased thyroid hormone levels in rats [42]. Therefore, it cannot be excluded that the physical stress associated with bear immobilization can cause a thyroid response unrelated to hibernation physiology. In hibernation, the bears were anesthetized in their dens, in close contact with humans. In the active state, the bears were chased and darted from a helicopter. It is unknown whether one of the two situations is more stressful to the bears than the other.

The specificity of the immunoassay used to quantify T_3_ and T_4_ in this study is not species dependent as the chemical structures of the thyroid hormones are identical between bears and humans. Moreover, the cross-reactivity with T_3_ and T_4_ analogues is low according to the manufacturer.

## Data Availability

The thyroid hormone dataset generated and analyzed in the current study is available from the corresponding author on reasonable request. The RNA sequencing dataset analyzed in the current study is available in the Genbank BioProject repository (Accession: PRJNA413091), https://www.ncbi.nlm. nih.gov/bioproject/?term = PRJNA413091.

## Abbreviations

ECLIA: Electrochemiluminescence immunoassay
FC: Fold-change
S: Summer/active
T_4_: Thyroxine
TBG: Thyroxine-binding globulin
TTR: Transthyretin
T_3_: Triiodothyronine
W: Winter/hibernating
